# Actinin-4 splice variant - a complementary diagnostic and prognostic marker of pancreatic neuroendocrine neoplasms

**DOI:** 10.7150/jca.37503

**Published:** 2020-02-10

**Authors:** Xiaojun Xu, Kazufumi Honda, Nami Miura, Shutaro Hori, Solange Le Blanc, Frank Bergmann, Matthias M. Gaida, Michael Volkmar, Simon Schimmack, Thilo Hackert, Oliver Strobel, Klaus Felix

**Affiliations:** 1 Department of General, Visceral and Transplantation Surgery, University Hospital Heidelberg, Heidelberg, Germany; 2 Department of Biomarkers for Early Detection of Cancer, National Cancer Center Research Institute, Tokyo, Japan; 3 Institute of Pathology, University Hospital Heidelberg, Heidelberg, Germany; 4 Surgery Division, Eiju General Hospital, Taito-ku, Tokyo, Japan; 5 Institute of Pathology, University Medical Center Mainz, Mainz, Germany; 6 Division of Molecular Oncology of Gastrointestinal Tumors, German Cancer Research Center (DKFZ), Heidelberg, Germany

**Keywords:** actinin-4, actinin-4 splice variant, pNEN, survival

## Abstract

**Introduction**: For pathological diagnosis of pancreatic neuroendocrine neoplasms (pNENs) the routinely used immunohistochemical markers are chromogranin A (CgA) and synaptophysin (Syn). Their ability as prognostic markers is not well established. A splice variant of actinin-4 (Actn-4sv) was recently found to be an excellent biomarker of neuroendocrine neoplasms of the lung. We aimed to investigate the expression of Actn-4sv in pNENs and evaluate its quality as a biomarker of pNENs.

**Methods**: Paraffin-embedded and frozen tissues specimens from 122 pNENs were analyzed. Western blots were performed to prove and compare the relative amount of Actn-4sv expression in pNENs tissue homogenates. For comparison pancreatic ductal adenocarcinoma (PDAC) and normal pancreatic tissues were analyzed in parallel. Immunohistochemistry (IHC) of paraffin sections of pNENs for Actn-4sv were performed and compared to the classic neuroendocrine markers CgA and Syn. Correlations were calculated between the staining intensity and distribution of Actn-4sv and staging, grading and afflicted lymph nodes respectively.

**Results**: Actn-4sv was expressed in 88.5% (108/122) of pNENs, but not in normal pancreatic tissues (0/14) or PDAC (0/14). Compared to CgA and Syn, Actn-4sv was not detectable in islet cells of the normal pancreas. Staining intensity of Actn-4sv on pNENs negatively correlated to the histological grading (Spearman r=-0.4990, p<0.0001) and staging (r = -0.2581, p = 0.0041) but no correlation to afflicted lymph nodes was found. A significantly better overall survival was observed for pNEN patients with higher expression of Actn-4sv (hazard ratio 2.7; log-rank test p= 0.0349).

**Conclusions**: The expression of Actn-4sv may be an important prognostic factor for patients with pNENs. Its expression correlates with the grading and staging of the tumors.

## Introduction

Pancreatic neuroendocrine neoplasms (pNENs) are rare tumors and comprising 1-2% of all pancreatic tumors 1. Neuroendocrine neoplasms including pancreatic NENs have recently gained attention due to growing incidence 2. The age-adjusted incidence of pNENs increased over the period 1973-2007 from 0.17 to 0.43 cases per 100,000 people in the USA 3. This increase is also due to increased physicians awareness and improvements in diagnostic imaging. Particularly the high sensitive and specific imaging techniques, such as computed tomography, SPECT with ^111^In-Pentetreotide and Positron Emission Tomography (PET) with ^68^Ga-DOTATATE, ^11^C 5-HTP and ^18^F-DOPA, multidetector-row CT and endoscopic ultrasound allow detecting and localizing pNENs 4, 5. PNENs occur with various symptoms, often related on the hormones produced. The molecular pathomechanisms are mostly unknown, but clinical studies showed that they may develop sporadic or are inherited 2.

PNENs can be divided into functional versus non-functional tumors with up to 85% being classified as non-functional 2. The majority of patients diagnosed with non-functional pNENs presents on admission with symptoms such as jaundice, weight loss, nausea, abdominal or back pain and pancreatitis, all symptoms with similar occurrence in patients with pancreatic adenocarcinoma 6. Functional pNENs are characterized by production and secretion of a variety of hormones including somatostatin, insulin, glucagon, serotonin, and pancreatic polypeptide. They are segregated according to the secreted hormone and the resulting clinical syndrome. From several studies it has been estimated that insulinomas are the most frequent functioning pNENs, followed by gastrinomas as the second most, whereas VIPoma and glucagonomas are rare.

PNENs are differentiated according to their mitotic activity, proliferation index, and growth pattern into neuroendocrine tumors (NET G1 - G2 - G3) and poorly differentiated neuroendocrine carcinomas (NEC G3) 7-9.

Easily detectable tumor markers, which are specifically expressed in pNEN-tissues and secreted into the blood circulation, are not established. Useful immunohistochemical tumor markers in tissue and serum peptides exist 10. A note of caution should be mentioned that routinely used markers for pathological identification of pNENs, chromogranin A (CgA) and synaptophysin (Syn), are also expressed in a variety of other tumor types, and in contrast are occasionally lost in pNENs 11, 12. CgA is a serum marker with a specificity of 85.7% and sensitivity of 67.9% and is routinely used as a histopathological marker on tissue 13-15. Because of the poor sensitivity and large inter-assay detection variance new tools for the diagnosis, prognosis, and monitoring of pNENs are of urgent need. Recent focus of research in this field is directed towards identification of biomarkers for biological targeted therapy and multiple marker test such as the NETest 16.

In 2004, Honda et al. discovered a splice variant (Actn-4sv) of the ubiquitously expressed actinin-4 17. By subsequent studies they suggested Actn-4sv as a prognostic marker for neuroendocrine tumors of the lung, the small cell lung carcinoma and the large cell neuroendocrine carcinoma 18. Actinin-4 is an actin-binding protein and component of the cytoskeleton. Originally it was associated with an elevated cell motility and tumor invasiveness 19. Further studies reported overexpression and the importance of actinin-4 in many malignant tumors as pro-tumor marker 20. The actinin-4 gene consists of two equally-long exons (8 and 8'), leading at transcriptional level to two different mRNAs, resulting in the exchange of the three amino acids N249G, A251L and S264C 17. The Exon 8 transcript was found ubiquitously expressed, however in healthy tissues the Exon 8' transcript called ”Actn-4sv” was found only in brain and testis and thus considered as a cancer testis antigen.

Okamoto et al. showed this spliced variant of actinin-4 as marker with strong diagnostic and prognostic validity in small cell lung carcinoma 21. Pathologically, this spliced-form occurs also in neuroendocrine tumors of other tissues, and the authors demonstrated RNAs for the variant actinin-4 (Actn-4sv) in all six investigated cell lines derived from NETs. However in pancreatic NEN the expression of the actinin-4 splice variant was not yet investigated.

Using frozen and FFPE pNEN-tissues we address the questions whether Actn-4sv may function as a potential indicator for pNEN, may be a complementary marker to CgA and Syn and whether the Actn-4sv levels are differently expressed in less differentiated versus high differentiated pNENs. We found that Actn-4sv is expressed in pNENs but not in normal pancreatic tissue. Additionally, we investigated the correlation between variant actinin-4-expression and factors such as outcome und invasive growth and found that patients with a high expression of Actn-4sv also have a better prognosis.

## Materials and methods

### Patients and samples

The study, performed at the Department of General Surgery, University of Heidelberg was approved by the Ethics Committee of the University of Heidelberg and written informed consent was obtained from all individuals from whom tissue samples were collected.

In this study, we retrospectively examined a collective of tissue samples of patients with pNENs from our database. Resected tumors were classified histopathologically according to the WHO and TNM classification and pNEN-patients with group I-IV staging were selected. The pNENs were diagnosed and classified using Ki-67 and the immunohistochemical markers CgA and Syn. In order to assemble a robust collective of pNEN samples the following procedure was used. At the beginning, all samples listed as neuroendocrine pancreatic neoplasms obtained between Feb. 2002 and Dec. 2012 in the Dept. of General Surgery, University of Heidelberg (n= 320) were acquired from our database (Biobank of the European Pancreas Center). The patients' samples were subsequently selected depending on the availability and amount of both frozen and corresponding FFPE pancreatic tissue (n= 176) at the time of the study. Prior analysis H&E staining was performed on all 176 pancreatic tissues and reviewed by experienced pathologists (F.B., M.M.G., and S.H.) to confirm disease diagnosis. Samples which have not fulfilled the quality criteria (e.g. with no significant amount of tumor, tissue alterations by electrothermic artifacts) were sorted out. Finally a total collective of 122 pNEN samples was obtained and all these samples were included into this study. Furthermore, 14 tissue samples of patients with PDAC, four with chronic pancreatitis (CP) and 14 normal tissue samples were randomly chosen and analyzed.

### Cell lines

The human pancreatic carcinoma cell lines AsPC-1, BxPC-3, CFPCA-1, MiaPaCa-2, PANC-1, SU86.86 and T3M4, and the human adenocarcinoma of the lung cell line A549 were obtained from ATCC (Rockville, MD, USA). The A549 transfected either with GFP or GFP- ACTN4va plasmids were generated to confirm the specific reactivity of anti-Actn-4sv antibody 18.

The cell lines cells were cultured in RPMI 1640 supplemented with 10% fetal bovine serum (FBS), 100 U/mL penicillin and 100 µg/mL streptomycin all from Life Technologies, Darmstadt, Germany. Cells were maintained in a humidified atmosphere with 5% CO_2_ at 37°C.

The human pancreatic neuroendocrine BON-1 cell line 22, 23 was obtained from Dr Modlin, Yale University School of Medicine, New Haven. The BON-1 cells were cultured in Hams's F12 and RPMI 1640 (1:1 v/v ratio) and as above supplemented with 10% FBS, 100 U/mL penicillin, and 100 µg/mL streptomycin at 37 °C in a humidified 5% CO_2_ atmosphere.

### Western blot

Western blot was chosen to prove the expression of Actn-4sv in pNENs. It was performed on tissue extracts from pNENs, PDAC, CP, normal pancreas, and cell lines of neuroendocrine tumor and exocrine pancreatic cancer. Tissue and cell extracts were obtained by first crushing the frozen tissues (60-80 mg/sample) while submerged in liquid nitrogen. The resulted powder was collected in 15 ml polypropylene tubes. Subsequently, 500 µl RIPA buffer (Tris 25 mmol/L, NaCl 75 mmol/L, NP-40 1%, CHAPS 250 mg/L, SDS 1%, pH 8.5) containing protease inhibitors (Roche, Mannheim, Germany) were added. Each tube was vigorously vortexed then shock-frozen in liquid nitrogen and stored overnight at -80 °C. The day after, using an ultrasonic homogenizer (SonoPuls mini20 Bandelin^®^, Berlin, Germany) the suspensions were subjected to a 30 sec. sonication step on ice (ampl. 80%, 0.99 kJ) and centrifuged at 16,000 × g for 10 min at 10 °C. Supernatants were collected and divided into aliquots, and the total protein concentration was determined using a Pierce BCA assay (ThermoScientific, Germany) and assessed on a plate reader (Multiskan EX, Thermo Scientific, USA).

Tissue- and cell-extracts were denaturated (5 min at 94°C), separated by SDS/Bis-Tris Gel 4-12% (NuPAGE, Life Technologies, Darmstadt, Germany) electrophoresis for 45 min at 200 V and blotted to a nitrocellulose transfer membrane (Whatman, Dassel, Germany). Each sample was incubated with the primary antibodies anti-Actn4sv (TransGenic Inc.,Kobe, Japan) clone 15H2 diluted 1:100 in TBSTM 17, 18, anti-pan-actinin-4 monoclonal antibody (ImmunoGlobe GmbH, Himmelstadt, Germany) diluted 1:2000 and mouse monoclonal antibody to β-actin (Sigma, Deisenhofen, Germany) diluted 1:8000 in 5 % BSA to control for equal loading. Then, 1x TBS and 0.1 % sodium azide (Calbiochem/Merck, Darmstadt, Germany) were added at 4°C overnight. In order to exclude interpretation mistakes and demonstrate that Actn-4sv is the sole antigen of immunoreactivity we tested the spliced variant peptide sequence used for antibody formation as competitive inhibitor submitted for commercial Synthesis to Peptide Synthesis Laboratories (PSL GmbH, Heidelberg, Germany).

After washing, membranes were incubated with a horseradish peroxidase-conjugated IgG (Santa Cruz, CA, USA) goat anti mouse or goat anti rabbit as secondary antibody diluted 1:5000 at 22°C for 30 min. With SuperSignal West Dura Extended Duration Substrate (Pierce, Waltham, USA) blot detection followed. The signals were recorded using a FUSION image acquisition system (Vilber Lourmat, Marne-la-Vallée, France). Band intensities were quantified using ImageJ software and normalized to the β-actin levels.

### Immunohistochemistry

The expression and semi-quantitative evaluation of Actn-4sv protein in pancreatic neuroendocrine neoplasms tissues was determined by immunohistochemistry (IHC). For comparative IHC analysis the expression of CgA, Syn, and wild type Actinin-4 were also analyzed.

The FFPE-tissues were cut into 4 µm thin slices and then deparaffinized to be subjected to immunohistochemistry (IHC). Staining was carried out using an automated slide stainer (AutostainerPlus, Dako, Denmark). The slides were incubated for half an hour at room temperature with anti-Actn-4sv monoclonal antibody (TransGenic Inc., Japan) (diluted 1:166 in antibody diluent Dako), anti-Synaptophysin monoclonal antibody (Millipore, USA) (1:100), anti-Chromogranin-A monoclonal antibody (Millipore, USA) (1:100) or anti-Actinin-4 monoclonal antibody (abnova, Taiwan) (1:8000). Negative controls were performed with Flex- Universal negative mouse IS759. Immunoreactivity was detected with the Immun-Star WesternC Chemilumindescent Kit (Bio-Rad, USA) using horseradish-peroxide reaction to produce the light signals. All slides were digitally scanned on a NanoZoomer-XR Digital slide scanner (Hamamatsu Photonics, Hamamatsu City, Japan).

The scoring algorithm was adapted from Hu et al., and had taken into consideration the proportion (percentage) of stained area of the tumor tissue and the intensity of the staining 24. Immunostaining of Actn-4sv less than 10% of tumor cells was denoted as negative (0). When >10% of tumor cells were stained, the expression was considered positive and denoted as weak (1), moderate (2) or strong (3) depending on the intensity. Scoring with (0) and (1) was categorized as the low expression, while scoring with (2) and (3) was categorized as the high expression. The results of IHC were judged by SH and FB. A randomly selected subset of 50 slides was being analyzed independently by MMG, there was a match of over 95%.

### RNAseq data analysis

Total RNA was isolated from randomly chosen fresh-frozen pNEN tissues using the RNA Plus Mini Kit (Qiagen, Hilden, Germany), according to the manufacturer's instructions. RNA quality was assessed on the Agilent 2100 Bioanalyzer using the Agilent RNA 6000 Nano Kit (Agilent Technologies, Waldbronn, Germany). Samples (n= 24) with a RNA-Integrity-Number (RIN) > 7.5 were used for RNA sequencing.

PNEN transcriptomes were generated from dual indexed libraries on an Illumina HiSeq4000 sequencer in paired-end, 100bp read-length mode by the DKFZ sequencing core facility. We used STAR 25 for mapping the raw data to the human reference genome (GRCh37), while subsequent read group addition and removal of duplicate fragments was done with Picard tools (http://broadinstitute.github.io/picard). The Cancer Genome Atlas (TCGA) pNEN transcriptomes were obtained from TCGA data portal as BAM files (mapped to hg19). The classification of the tumors was verified from the provided pathology reports. RNA-seq datasets of PDAC cell lines were downloaded from the TCGA legacy archive.

To determine the relative expression of the alternative *ACTN4* exons 8 and exon 8' 17 spliced into the Actn-4 and Actn-4sv mRNAs, respectively, we used BEDtools 26 to extract the mean coverage of both exons. Normalization was performed by dividing the mean coverage of exon 8 and 8' to the number of reads in the mapped, not duplicated transcriptome and multiplication of the results by 1 million. For plotting the results, we used the R package 'ggplot2' 27.

### Biostatistics

Statistical analysis and graphical data presentation were performed using GraphPad Prism 5 (GraphPad Software Inc., San Diego, CA) and SAS software (Release 9.4, SAS Institute, Inc., Cary, NC). The survival rates were assessed using the KaplanMeier method. Patients alive at the last follow-up were censored. In all graphs, overall survival (OS) is defined as the time from the date of the operation to either death from any cause or last follow-up. The difference between the Kaplan-Meier curves was tested for significance applying the log-rank test. Differences were considered to be statistically significant at P < 0.05.

Biometric analysis was performed to examine the strength of correlation between the clinical parameters including age staging, grading, lymph node metastasis and survival with levels of IHC-based validations of the Actn-4sv. Depending on the character of the distributions of the quantitative parameters in each group, the correlation coefficient r with its corresponding p-value of Pearson correlation was used to analyze the correlations. The character of the distributions of the quantitative parameters was determined using the Shapiro-Wilk test and a normal probability plot.

## Results

### Patient clinico-pathological characteristics

We evaluated pNEN samples from 122 patients together with 14 tissue samples of patients with PDAC, four CP samples, and 10 normal tissue samples as controls. PNEN-patient demographics are summarized in Table 1.

### Expression of actinin-4 splice variant in pNEN

Tumor associated antigens, which are selectively overexpressed in pNEN cells, particularly already at early stages, are ideal target proteins for early detection and tumor monitoring. For this purpose we investigated an isoform of Actinin-4, the so called Actn-4sv, for screening and risk stratification of pNEN. Prior to large scale experimental analysis we proved the expression of actinin-4 and its variant Actn-4sv as eligible markers for pNENs. 14 human pNEN-tissue extracts and corresponding donor pancreatic tissues extracts, 10 PDAC, four CP as well as two normal pancreatic tissue lysates were tested for the expression of Actinin-4 and Actn-4sv protein levels. The tissue extracts were subjected to PAGE and subsequently immunoblotted to monitor actinin-4 or its isoform Actn-4sv, the latter through use of a monoclonal antibody (15H2) that reacted specifically with a peptide sequence (DIVGTLRPDEKAIMTYVSC) derived from the variant actinin-4 protein, but not with the corresponding sequence of the ubiquitous protein (DIVNTARPDEKAIMTYVSS). To assess specificity and exclude interpretation mistakes we tested the spliced variant peptide sequence used for antibody formation as competitive inhibitor and the western blots showed no bands indicating Actn-4sv as sole antigen.

**Figure 1:**
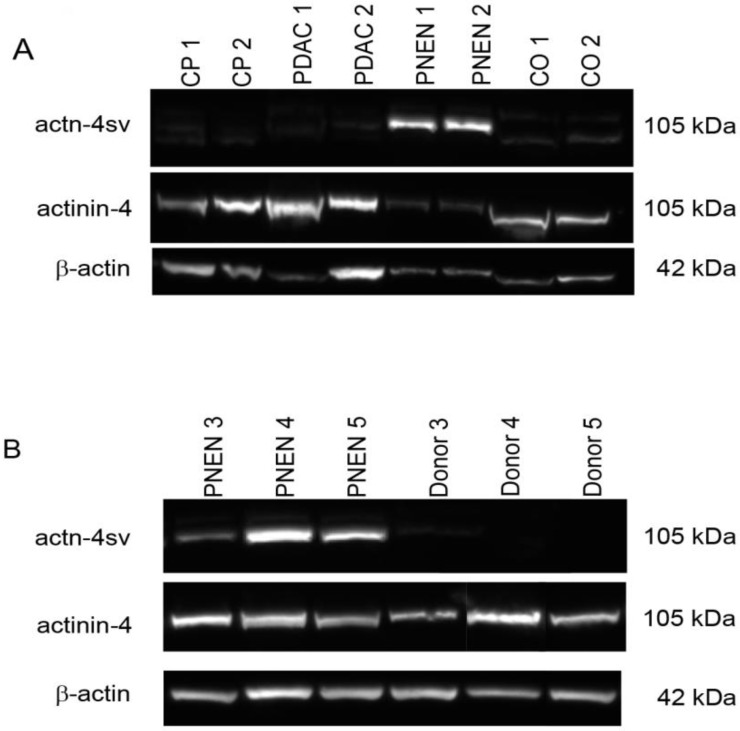
Expression of Actinin-4 and actinin4-splice variant (Actn-4sv) in pancreatic tissue extracts from chronic pancreatitis (CP), pancreatic ductal adenocarcinoma (PDAC), pancreatic neuroendocrine neoplasia (pNEN), and normal tissue using western blot. **A**: Lanes 1-2: CP-, lanes 3-4: PDAC-, lanes 5-6: pNEN- and lanes 7-8: normal-pancreatic (CO) tissue extracts. **B:** Detection of Actn-4sv in three pNENs but not in the corresponding adjacent normal tissues (donor). Moderate to strong expression of Actn-4sv was detectable in all pNEN tissue extracts but not detectable in normal pancreatic tissue, CP and PDAC extracts. Using a pan-actinin-4 antibody, detecting both the wild type actinin-4 and Actn-4sv, positive staining was found in all lysates. Immunoblots for β-actin were used as loading control reference.

A representative western blot is presented in Figure 1 A. The Actn-4 spliced form was not detectable in CP, PDAC and normal control samples. On the other hand Actn-4sv was detected as an approximately 105 kDa band in all pNENs, however with different intensities as shown in Figure 1A-B. The normal pancreatic tissues were negative for Actn-4sv. A semiquantitative analysis applying ImageJ and β-actin as a reference protein revealed a range of actn-4 sv/β-actin ratio between 0.5-1.4. Using a pan specific-Actinin-4 Ab which binds to the N-terminal part (MGDYMAQEDDW) of the protein, a 105 kDa band was found in all lysates.

### Expression of actinin-4 and actn-4 splice variant in cell lines

In parallel to the tissue extracts cell lysates from AsPC-1, BxPC-3, CFPAC-1, MIA PaCa-2, PANC-1, SU8686, T3M4 and BON-1 were also subjected to PAGE and immunoblotted to monitor the Actinin-4 and its isoform Actn-4sv. As internal control the adenocarcinoma of the lung cell line A549 transfected with Actn-4sv tagged with GFP-protein was used to confirm the reactivity of anti-actn-4sv antibody 18.

**Figure 2:**
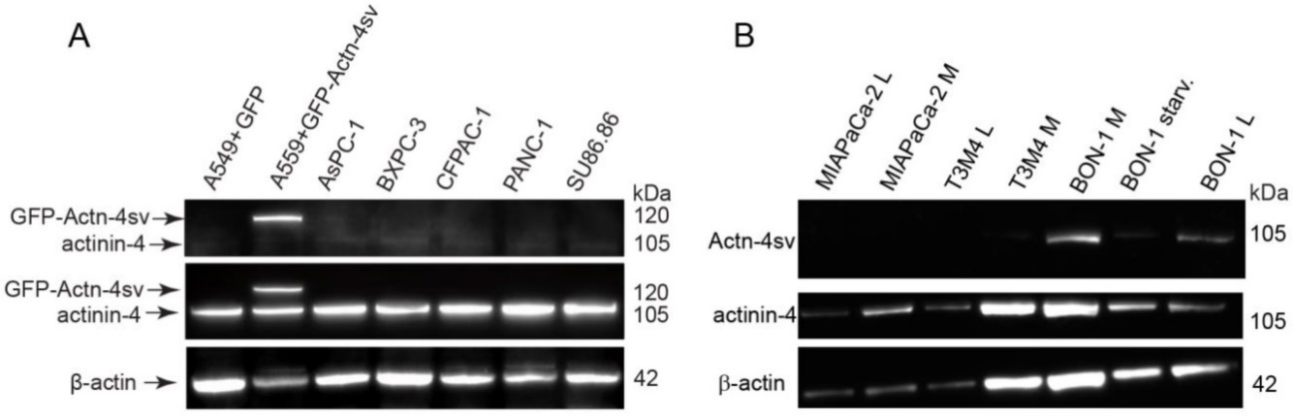
Expression of Actinin-4 and Actn4sv proteins in human PDAC cell lines and human pNEN cell line BON-1. **A:** Proteins were extracted from A549 cells that had been transfected (mock) with plasmid pEGFP (lane 1) or pEGFP-ACTN-4-SV (lane 2) and PDAC cell lines (lane 3-7) and blotted with 15H2 (Actn-4sv), 13G9 (pan-ACTN-4) and anti-β-actin (loading control) antibodies. **B:** Actn-4sv expression was only detectable in BON-1 cells. Lanes 1- 2: MiaPaca-2; lane 3-4: T3M4; lane 5: BON-1 (M); lane 6: BON-1after 72 h starvation (low glucose, serum free), lane 7, BON-1 (L). (L= low glucose 5.5µM and M = medium glucose 22 µM).

The specific reactivity of anti-actn4-va antibody 15H2 is clearly visible as a single band in GFP-Actn-4sv transfected A549 cells but absent in mock transfected (GFP) as shown in lane 1 and lane 2 as quality control Fig. 2A. Using the pan-actinin-4 Ab 13G9 the protein was detected in all cell lines. However, Actn-4sv was only clearly visible in the pNEN cell line BON-1 as shown in Figure 2B. Withdrawal of nutrition “starvation” for 72 h revealed no significant effect on Actn-4sv expression.

### Expression of Chromogranin A, Synaptophysin, and actinin-4 splice variant in pNENs determined by IHC

It is well known that protein overexpression of Actinin-4 is a prognostic biomarker for invasive PDAC of the pancreas 28. Here we investigated the protein expression of Actinin-4 and its variant Actn-4sv in pNENs and compared it to normal pancreatic tissue using IHC.

**Figure 3:**
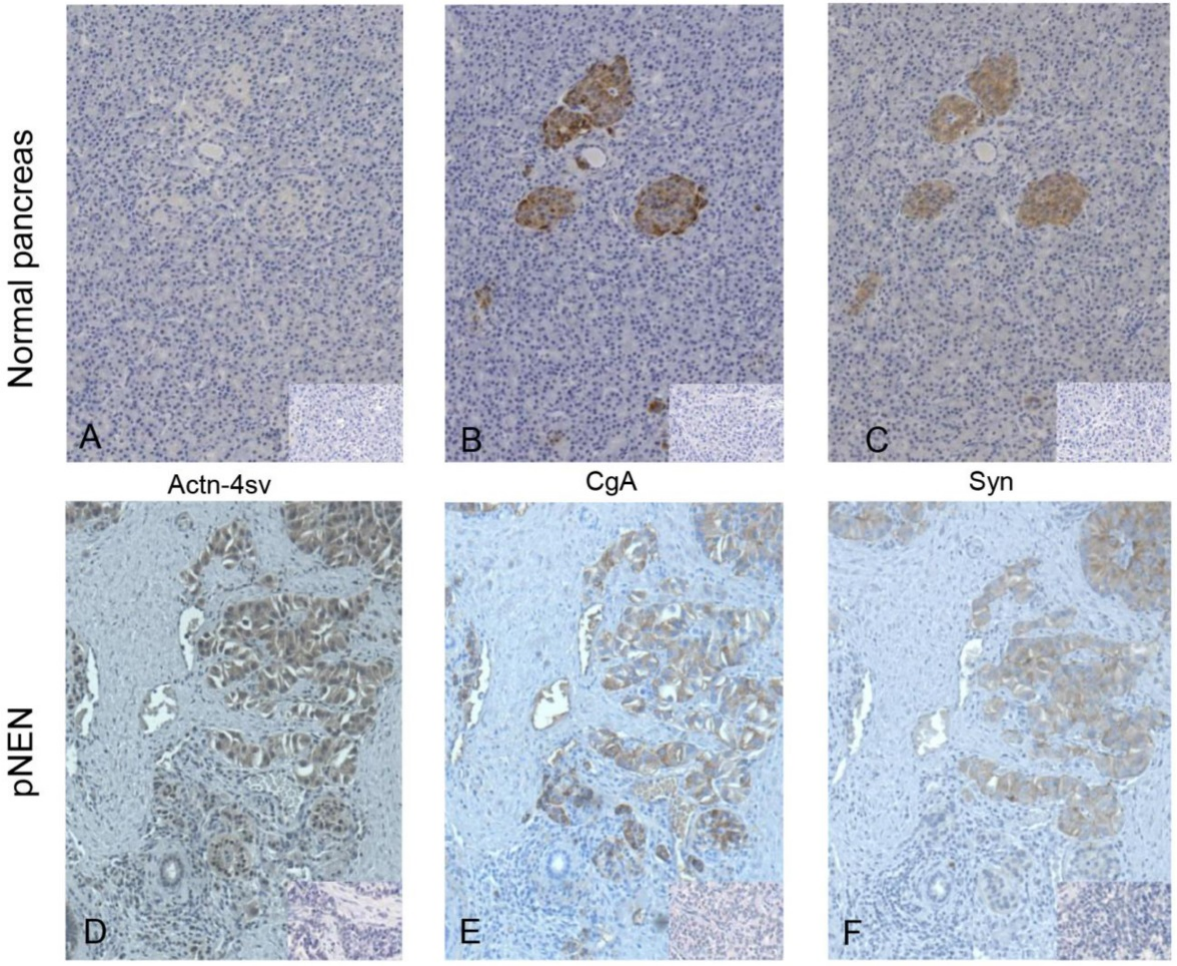
IHC of FFPE tissue on consecutive slides of normal pancreatic tissue (panel A-C) and pNEN (panel D-F), small inserts show the corresponding negative controls. Staining for Actn-4sv (A, D), CgA (B, E), and Syn (C, F). In normal pancreas the islet cells are stained for CgA and Syn but not for Actn-4sv. In contrast in pNEN a strong staining for the three markers is visible. (Magnification x 200)

IHC revealed staining of normal pancreatic endocrine tissue for CgA and Syn, but no staining for Actn-4sv was detectable as shown in Figure 3 A-C. In contrast to nornal tissue a strong expression of the Actn-4sv was observed in pNENs (Figure 3D).

After optimization of staining conditions on the automated slide stainer a large cohort of pNEN composed of 122 patients was assessed for the expression and distribution of Actn-4sv, and compared to the expression of the reference markers CgA and Syn. The results are summarized in Table 2. A positive Actn-4sv staining was obtained for 108 pNEN tissue samples (88.5%). The samples were then divided into two categories according to the staining intensity: low intensity comprising faint to weak staining and high intensity comprising moderate to strong tissue staining. A variation in stained tumor area was also observed and subdivided into five groups as presented in Table 2.

### Correlation between intensity of Actn-4sv staining and grading and staging

**Figure 4:**
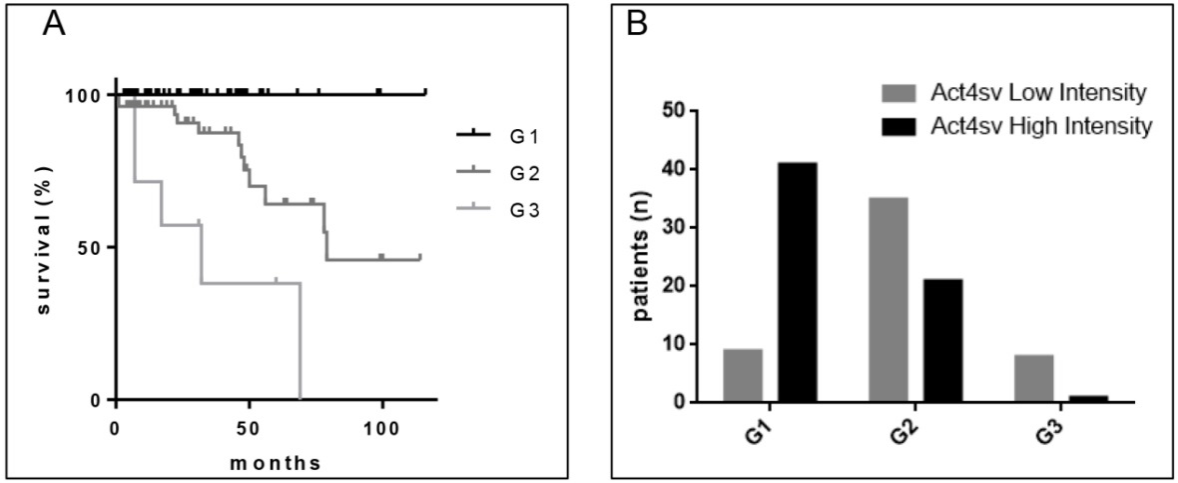
** A**: Survival of pNEN patients in regard to grading. **B:** Correlation of Actn-4sv staining intensity vs. grading.

Because tumor grade is considered an important prognostic variable for survival as shown for our analyzed cohort in Figure 4A, we assessed the Actn-4sv expression in correlation to pNEN grading (Figure 4B). The tumor grade was available for 120 pNEN patients, and the distribution was as follows: for NET G1, n = 50 patients; for NET G2, n = 61 patients; for NET G3, n = 7 and NEC (G3) large cell type n = 2 patients. Protein expression (staining-intensity) of Actn-4sv statistically negatively correlated with the grading (Spearman, r = -0.4990, P < 0.0001).

As the next step we analyzed the statistical correlations between Actn-4sv expression and staging. Staging according the UICC 2009 was available for 86 patients and the majority of pNEN were stage IIIB (n= 33) and stage IV (n= 29). Also a correlation of Actn-4sv staining-intensity and tumor staging (r= -0.2581, P= 0.0041) was found. However, there was no correlation between Actn-4sv protein expression and lymph node metastasis. The results are summarized in Table 3.

### Survival of patients in regard to intensity of actinin-4 splice variant staining

**Figure 5:**
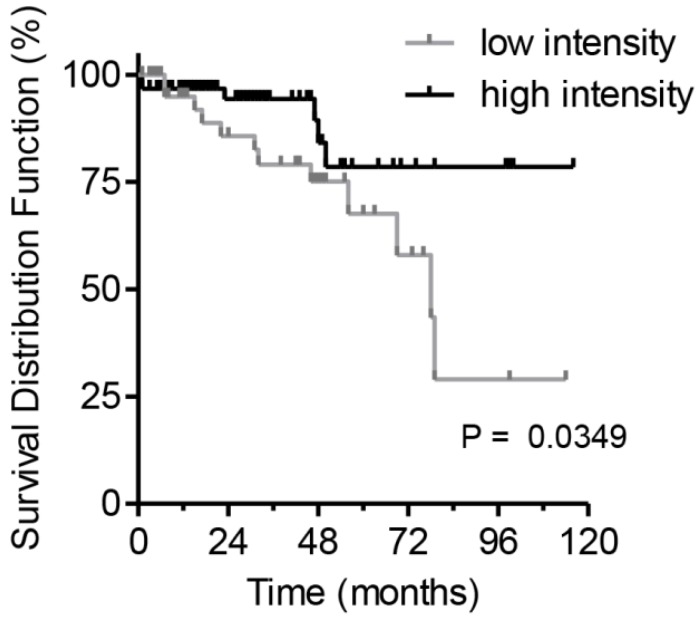
Postoperative survival of pNEN patients in regard to Actn-4sv expression (Kaplan-Meier estimates). Significantly better survival for patients with median to strong Actn-4sv staining intensity (log-rank test, p = 0.0349).

To estimate the clinical prognostic significance of Actn-4sv expression, Kaplan-Meier survival analysis and a log-rank test were performed. The analysis was performed on survival data available for 122 pNEN patients. A staining intensity cut-off of <50% was selected for evaluating the Actn-4sv as a marker of overall survival (OS). For all 122 PNEN analyzed, a statistically significant difference in OS was found. Median to strong Actn-4sv staining intensity (**≥** 50% n = 70) was prognostic for longer OS, compared to 52 patients with Actn-4sv staining intensity (faint to low) below 50% (log-rank p = 0.0349) as shown in Figure 5.

**Figure 6:**
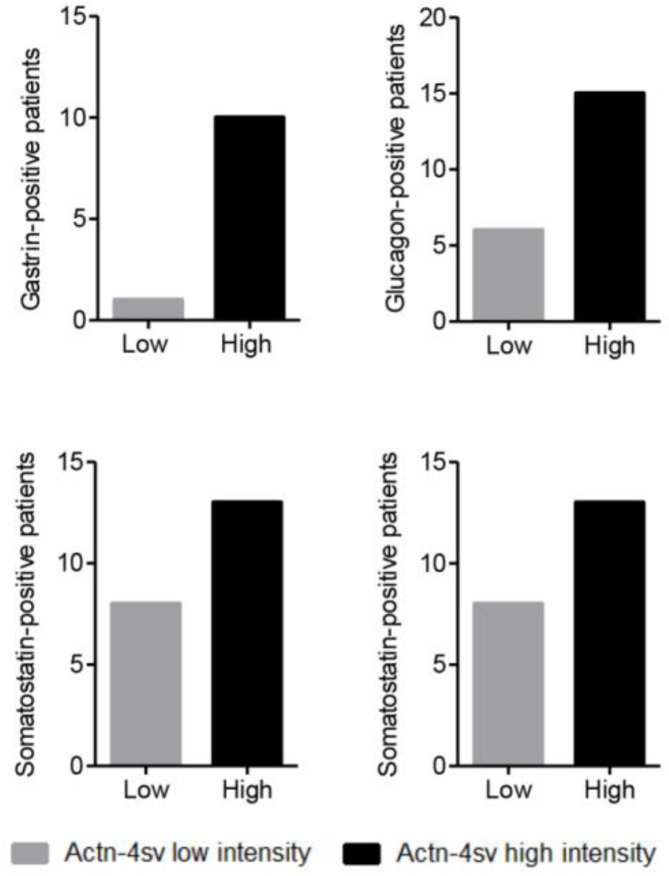
IHC on tumor tissues for expressed hormones (positive/negative): insulin (27/75), gastrin (11/77), glucagon (21/78) and somatostatin (21/74) versus intensity (Low High) of Actinin-4 splice variant staining.

As the next part of the study we investigated the Actn-4sv expression in relation to pNEN expressing hormones. As presented in table 1 the entire PNEN-cohort revealed several hormone expressing tumors: 27 insulin-positive, 11 gastrin-positive, 21 glucagon-positive and 21 somatostatin-positive of which several were double- or even triple-positive hormone-producing tumors. Figure 6 summarize the Actn-4sv staining intensity in pNEN expressing hormones.

**Figure 7:**
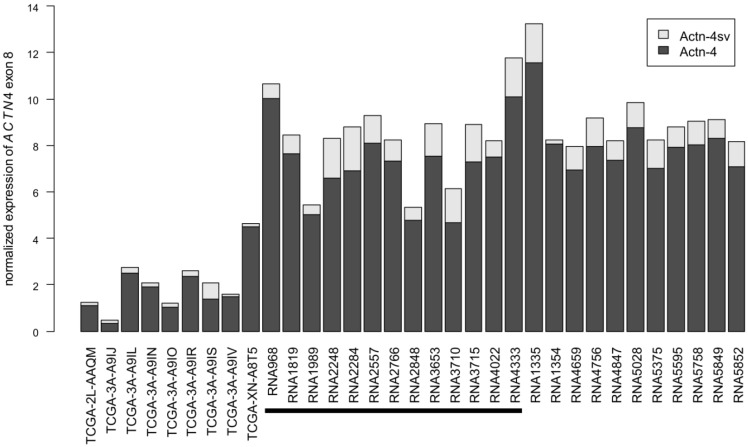
** Abundance of Actn-4 and Actn-4sv mRNA variants in pNEN transcriptomes.** RNAseq datasets from TCGA (left) and generated in-house were analyzed for incorporation of exon 8 (Actn-4, dark grey) or the alternative exon 8' (Actn-4sv, light grey). All samples analyzed express Actn-4sv at RNA level. For comparability between samples, expression was normalized as described in the Materials & Methods section. The black horizontal bar denotes those samples for which also Actn-4sv IHC has been analyzed in this study.

To assess the expression of Actn-4sv on RNA level, we analyzed the utilization of the alternative exon 8'^17^ in relation to the exon 8 incorporated into Actn-4 mRNA. To this end, we obtained pNEN transcriptomes from TCGA and analyzed a more comprehensive set of in-house pNEN transcriptomes. *ACTN4* gene expression varies between samples and all analyzed samples express Actn-4 splice variant mRNA (Figure 7). Actn-4sv comprises, on average, 13% and ranges between 2% (RNA1354) and 34% (TCGA-3A-A9IS) of the total *ACTN4* mRNA.

However a correlation between the Actn-4sv protein expression assessed by IHC (n= 13) and the corresponding splice variant Actn-4sv mRNA could not be established, likely due to different tissue sections (FFPE vs frozen) of the tumor used for IHC and RNA.

**Figure 8:**
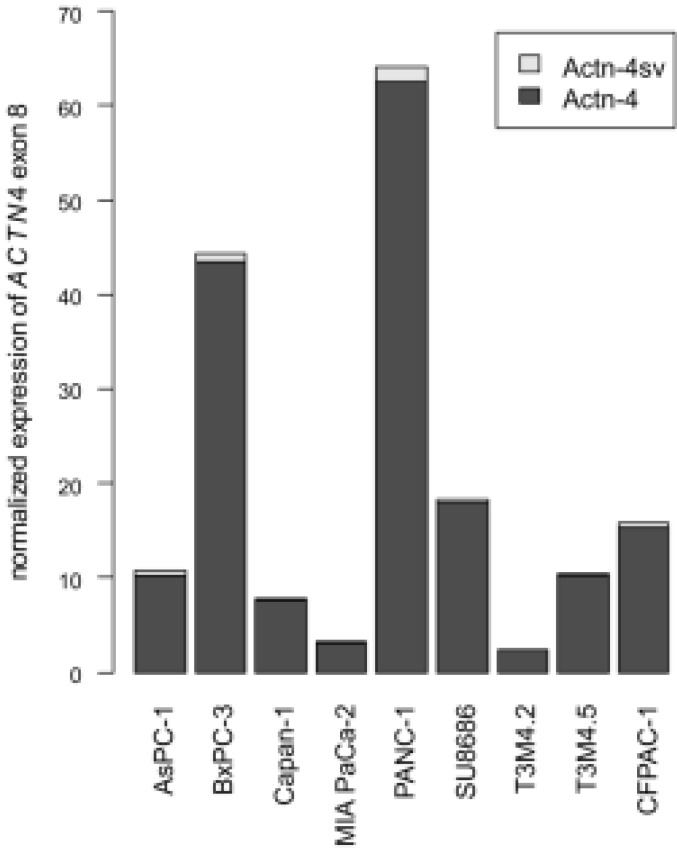
** Abundance of Actn-4 and Actn-4sv mRNA variants in PDAC cell lines.** RNAseq datasets of pancreatic ductal adenocarcinoma cell lines were obtained from the TCGA Legacy Archive and analyzed analogously to the pNEN transcriptomes. ACTN4 expression varies widely between cell lines while Actn-4sv (light grey) expression is generally low, ranging between 1 and 5% of total ACTN4 mRNA.

Additionally, we assessed Actn-4sv mRNA expression in transcriptome data of PDAC cell lines (Figure 8). Here, the Actn-4 splice variant is universally expressed at low levels between 1 and 5% (mean=3%).

## Discussion

The establishment of a reliable marker to assess the malignant behavior of pNENs would be very important to optimize the patients' therapy. Neuroendocrine proteins CgA, Syn and the neural cell adhesion molecule CD56 are the conventional primary diagnostic markers in pNENs. Additionally molecular markers such as, Ki-67, CK19, p27, p21, p53, cyclin D1, Bcl-2, E-cadherin, and vimentin are increasingly used to predict patient outcome. Antibodies have been raised against mutant-proteins for immunoassays, and immunohistochemistry has been found to be a fast and efficient method for evaluating the molecular aberrations on protein level 29. These aberrant-specific antibodies specifically bind to altered regions of the proteins encoded by the mutated genes or alternatively spliced transcripts but do not bind to the wild-type proteins.

In this study we investigated the expression of the spliced transcript Actn-4sv in pancreatic neuroendocrine neoplasms and compared it to the expression of CgA and Syn which are standard diagnostic markers for the pathological diagnosis and immunohistochemical confirmation of pNENs 30. The Actn-4sv marker was chosen based on previous reports suggesting his prognostic value in other NENs. Here we demonstrated, first by western blotting, the presence of Actn-4sv in all analyzed pNEN tissue-lysates. This variant protein was undetectable in extracts derived from PDAC-, CP- and pancreatic donor-tissues. Subsequently, applying IHC we analyzed a larger cohort of pNENs and confirmed the expression of Actn-4sv in the resected tissues sections. Our data demonstrate that the Actn-4sv is expressed at the protein level in 86% of the pNENs, suggesting Actn-4sv may be a useful diagnostic biomarker for pNENs or complementary marker in cases where CgA and Syn perform poorly. Immunoblotting and IHC are convenient approaches and frequently used methods for assessment of protein amount and distribution in tissue lysates and histological sections. Nevertheless, data must be interpreted with caution as the antigen-antibody complex formation on a membrane or on a tissue matrix surface may be accompanied by unspecific binding interactions. In order to exclude interpretation mistakes we tested the spliced variant peptide sequence used for antibody formation as competitive inhibitor. These western blot and IHC data revealed the Actn-4sv as sole antigen of immunoreactivity.

The role of Actinin-4 in cancer and particularly its involvement in tumor progression and metastasis is well documented for many cancers including colorectal, lung, gastric, cervical cancer, and PDAC as reviewed in 20. The overexpression of Actinin-4 which is based on the gene amplification of *ACTN4* has been observed in invasive PDAC and these patients showed a worse prognosis for overall survival than those with weak Actinin-4 expression 31.

The variant transcript Actn-4sv was reported to be expressed under normal conditions only in human testis and in trace amounts in brain tissue but was not detected in any other normal organs 17. The current knowledge about the Actn-4sv function and role in cancer is limited 17, 18, 21 and no reports on Actn-4sv protein in pancreatic NENs are known.

Our pNEN transcriptome analysis for incorporation of exon 8 or the alternative exon 8' into the *ACTN4* mRNA applying the RNAseq datasets from TCGA and our in-house generated, revealed that both forms the ubiquitous Actinin-4 and the splice variant Actn-4sv are expressed on the mRNA level (ranging between 2-34% in relation to exon 8). Interestingly, the analogous analysis of *ACTN4* expression in PDAC cell lines showed not only larger transcriptome differences between analyzed cell lines but also a much lower Actn-4sv expression, ranging between 1 and 5% of total *ACTN4* mRNA.

Here we report for the first time the protein expression of this alternatively spliced actinin-4 in pNENs but not in non-neoplastic pancreatic islets. This is in line with previous reports on Actn-4sv in high grade neuroendocrine pulmonary tumors (HGNTs) 18. However, in contrast to HGNTs where the expression of Actn-4sv was significantly associated with poorer overall survival, in the pNENs the opposite observation was made. The overall survival of patients with moderate to strong Actn-4sv protein expression was significantly better than that of patients with faint or weak Actn-4sv expression which revealed an unfavorable postsurgical outcome. Our result of increased Actn-4sv expression being a better survival of the patients is also consistent with our finding that increased Actn-4sv staining correlates with better grading and tumor staging of the pNENs. Therefore we assume that neuroendocrine pancreatic neoplasms have a fundamentally different interaction with Actn-4sv on a molecular basis than neuroendocrine tumors of the lung.

Nikopoulos et al. showed that a decreased Actinin-4 expression favor cell adhesion abnormalities and metastatic ability 32. Assuming that Actn-4sv, as a subgroup of Actinin-4, performs similar functions and thus stronger expression of Actn-4sv prevents cell adhesion abnormalities, then our data demonstrating that increased Actn-4sv expression in the pNENs correlates with a better outcome for the tumor patients are in line with the finding of Nikopoulos et al. work. Additionally, since Actinin-4 can be localized in the cell membrane, cytosol and also the nucleus its subcellular distribution may influence the tumor cell properties. In breast tumors and few cell lines Actinin-4 was detected immunohistochemically in cytosol and nucleus, and cells with cytoplasmic expression were more motile and metastatic 19. Likely subcellular Actn-4sv distribution has also effects on motility of the pancreatic neuroendocrine tumor cells because in few of our pNENs a staining for Actn-4sv was only nuclear or cytoplasmic. Further research has to be conducted to show possible linking between intracellular location and outcome of patients. It is conceivable that nuclear or cytoplasmic location of Actn-4sv may be correlated to tumor invasion and metastasis. Our preliminary data on the subcellular localization/distribution of Actn-4sv in context of migration and invasiveness of BON-1 and QGP-1, two cell lines derived from human pNETS indicate that factors such as hypoxia and nutrient deprivation might influence the migratory behavior (results not shown).

It is also noted that Actn-4sv is not found in the serum, so it is not suitable as serum marker. At a high sensitivity of 88.5 % it may be useful in clinical setting to combine this marker with a serum marker such as CRP, which was described as an independent prognostic marker by Wiese et al. 33.

## Figures and Tables

**Table 1:** Pancreatic neuroendocrine neoplasia patient characteristics

Patients	n = 122	
	n	%
**Gender**		
female	53	43.4
male	69	56.6
**Age**		
median	56 (range	12 - 85)
< 50 y	50	41.0
≥ 50 y - < 65 y	41	33.6
≥ 65 y	31	25.4
Grading*		
NET G1	50	41.0
NET G2	61	50.0
NET G3	7	5.8
NEC G3 small cell type	0	--
NEC G3 large cell type	2	1.7
n/a	2	1.7
**Hormone expression (pos./neg)**		
insulin	(27/75)	
gastrin	(11/77)	
glucagon	(21/78)	
somatostatin	(21/74)	
**Resection**		
R0	88	72.1
R1	16	13.1
R2	18	14.8
Metastasis		
M0	96	31.6
M1	26	68.4
**Lymph nodes**		
pN0	73	59.9
pN1	49	40.1

*WHO classification 2017

**Table 2:** Immunohistochemistry staining for chromogranin A (CgA), synaptophysin (Syn) and actinin-4 splice variant (Actn-4sv).

Immunohistochemistry	patients	
	n	%
**PNENs**	122	100
**CgA**	120	98.4
**Syn**	120	98.4
**Actn-4sv intensity**	108	85.5
negative - weak intensity: Low	52	42.6
moderate - strong intensity: High	70	57.4
**Actn-4sv stained tumor area**		
<10%	14	11.5
10-39%	7	5.7
40-59%	12	9.8
60-79%	30	24.6
80-100%	59	48.4

**Table 3:** Correlation between Actn-4sv staining intensity and staging and grading.

Correlation between	Result	r	P*
Intensity vs. grading	yes	-0.4990	<0.0001
Intensity vs. staging	yes	-0.2394	0.0264
Grading vs. staging	yes	0.2352	0.0346
Intensity vs. lymph nodes**	no		

*Spearman, r=-0.4990, *P*<**** and staging (r=-0.2394, *P*=*), but not with affected lymph nodes. **Positive lymph nodes out of all examined lymph nodes.
